# Association between inflammation and systolic blood pressure in RA compared to patients without RA

**DOI:** 10.1186/s13075-018-1597-9

**Published:** 2018-06-01

**Authors:** Zhi Yu, Seoyoung C. Kim, Kathleen Vanni, Jie Huang, Rishi Desai, Shawn N. Murphy, Daniel H. Solomon, Katherine P. Liao

**Affiliations:** 1 Department of Epidemiology, Johns Hopkins Bloomberg School of Public Health, Baltimore, MD USA; 2 Department of Biostatistics, Johns Hopkins Bloomberg School of Public Health, Baltimore, MD USA; 3 Division of Rheumatology, Allergy and Immunology, Brigham and Women’s Hospital, Boston, MA 02115 USA; 4 Division of Pharmacoepidemiology and Pharmacoeconomics, Brigham and Women’s Hospital, Boston, MA USA; 5 Research Computing, Partners HealthCare, Charlestown, MA USA; 6 Laboratory of Computer Science, Massachusetts General Hospital, Boston, MA USA; 7 Department of Biomedical Informatics, Harvard Medical School, Boston, MA USA

**Keywords:** Inflammation, Blood pressure, Rheumatoid arthritis

## Abstract

**Background:**

The relationship between inflammation and blood pressure (BP) has been studied mainly in the general population. In this study, we examined the association between inflammation and BP across a broader range of inflammation observed in rheumatoid arthritis (RA) and non-RA outpatients.

**Methods:**

We studied subjects from a tertiary care outpatient population with C-reactive protein (CRP) and BP measured on the same date in 2009–2010; RA outpatients were identified using a validated algorithm. General population data were obtained from the National Health and Nutrition Examination Survey (NHANES) as comparison. To study the cross-sectional association between CRP and BP in the three groups, we constructed a generalized additive model. Longitudinal association between CRP and BP was examined using a repeated-measures linear mixed-effects model in RA outpatients with significant change in inflammation at two consecutive time points.

**Results:**

We studied 24,325 subjects from the outpatient population, of whom 1811 had RA, and 5561 were from NHANES. In RA outpatients, we observed a positive relationship between CRP and systolic BP (SBP) at CRP < 6 mg/L and an inverse association at CRP ≥ 6 mg/L. A similar inverse U-shaped relationship was observed in non-RA outpatients. In NHANES, we observed a positive relationship between CRP and SBP as demonstrated by previous studies. Longitudinal analysis in RA showed that every 10 mg/L increase in CRP was associated with a 0.38 mmHg reduction in SBP.

**Conclusions:**

Across a broad range of CRP observed in RA and non-RA outpatients, we found an inverse U-shaped relationship between CRP and SBP, highlighting a relationship not previously observed when studying the general population.

**Electronic supplementary material:**

The online version of this article (10.1186/s13075-018-1597-9) contains supplementary material, which is available to authorized users.

## Background

Inflammation is associated with elevated blood pressure (BP) in the general population [1, 2]. In rheumatoid arthritis (RA), the levels of inflammation, as measured by C-reactive protein (CRP) can be 10-fold higher than in the general population. Whether the relationship between inflammation and BP remains the same under these conditions has not been closely studied [3, 4]. Studying the association between inflammation and BP is particularly important in RA; cardiovascular (CV) risk is 1.5–2.0 times higher compared to individuals from the general population of similar age, sex, and CV risk factors [5–7]. This excess risk is attributed to inflammation [8].

There is also increasing evidence that inflammation may modify traditional risk factors such that higher levels of inflammation are associated with lower cholesterol levels [9–11]. However, these lower cholesterol levels are not associated with reduced CV risk; in RA, any given level of cholesterol may confer a higher CV risk than expected based on general population-based studies due to the added effect of inflammation [10, 12]. Few studies have examined closely the relationship between inflammation and BP levels, and whether a similar modification may be occurring. Since both inflammation and BP are associated with an increased risk of cardiovascular disease (CVD) [13–16], understanding the relationship between the two can provide insight into pathways linking inflammation and CV risk not only in RA, but also in the general population.

The objective of this study was to examine the association between inflammation and BP in RA compared to a non-RA outpatient cohort, and a general population cohort. We hypothesize that the relationship between inflammation and BP may differ at the higher levels of inflammation observed in RA and other inflammatory conditions compared to levels observed in the general population.

## Methods

### Study populations

#### General population cohort

The National Health and Nutrition Examination Survey (NHANES) served as the general population control for this study [17]. The most recent NHANES data on CRP and BP spans from 1 January 2009 to 31 December 2010. Thus, this time frame was used to select data for all subjects in this study. Additionally, we studied only subjects aged 18 years or older.

#### Outpatient population

To obtain a broader range of CRP values, we examined the data on all outpatients followed at two large tertiary hospitals, Brigham and Women’s Hospital (BWH) and Massachusetts General Hospital (MGH), Boston, MA, USA, with clinical data on approximately 7.2 million patients. BWH and MGH share one electronic medical record (EMR) system. The Partners Research Data Repository (RPDR) [18] enabled efficient extraction of de-identified clinical data across the two institutions. Using the same inclusion criteria applied to NHANES, the outpatient population consisted of adult patients aged 18 years and older, who had CRP and BP measured on the same day at an outpatient visit between 1 January 2009 and 31 December 2010. For the primary analysis we used the first same-day CRP and BP measurements of each subject. Inpatients were not included to reduce potential confounding from severe conditions such as sepsis.

#### RA outpatient population and non-RA outpatient population

We defined a subset of the outpatient population with a validated diagnosis of RA, identified using a validated and published algorithm with a positive predictive value of 94% [19, 20], as the RA outpatient population. For this cohort, we additionally extracted longitudinal data on subjects who had a change in CRP ≥ 10 mg/L between two time points and also had BP measurements at the two time points. We defined the rest of the outpatient population, subjects not diagnosed with RA, as the non-RA outpatient population.

### CRP and BP measurements

In the NHANES cohort, CRP was measured using a high-sensitivity assay with latex-enhanced nephelometry [21]. Three consecutive BP measurements were obtained by certified examiners using a sphygmomanometer after participants had rested quietly while sitting for 5 min [22]. For the analysis, we only included subjects with valid BP readings for all three attempts. In the outpatient population, CRP was measured using the high-sensitivity CRP assay, performed in the clinical laboratories at Partners Healthcare using standardized methods [23]. BP is a required measurement at all outpatient visits at Partners Healthcare, obtained by trained healthcare providers, and entered as structured data into the EMR. BP values are recorded as systolic blood pressure (SBP) and diastolic blood pressure (DBP).

### Statistical analysis

For the primary analysis, we focused on the association between CRP and SBP because it is the main target of BP intervention studies of CVD risk [13]. The CRP levels were common-log-transformed to normalize the distribution. Using graphical techniques, we visualized the relationship between CRP (common-log-transformed) and SBP in the RA outpatient population. From this graph, we observed that the relationship was non-linear. Thus, we constructed a generalized additive model with penalized splines as the approach to study the association between CRP and SBP, adjusting for age (continuous), gender, and race (non-Hispanic white/non-Hispanic black/other races); electronic prescription data were used to obtain information on anti-hypertensive medication, statins, methotrexate (MTX), and tumor necrosis factor inhibitors (TNFi), as a binary value for ever/never use. Since we are interested in studying a non-linear association between the variables, we applied the generalized additive model. This approach uses semi-parametric methods to enable modeling of non-linear association [24]. Additionally, we tested the associations between CRP and DBP, pulse pressure (PP), and mean arterial pressure (MAP) using the same model. The same methods were also applied to the non-RA outpatient population and NHANES.

In the RA outpatient population, we performed a longitudinal study examining the association between the changes in inflammation and changes in BP. In RA, having a high level of inflammation is typically a temporary state due to an RA flare or inadequate response to treatment. Thus, subjects with RA routinely have large changes in CRP in the course of routine care, providing an opportunity to study the association between longitudinal changes in inflammation and BP. For this study, we focused on patients with RA who had an increase or decrease in CRP by ≥ 10 mg/L, which is considered a significant change in inflammation [25, 26]. We applied linear mixed effect models to account for correlation between repeated measures within subjects to examine the associations between changes in CRP and changes in SBP. We defined baseline as the day of their first same-day CRP and BP measurements during the study period. Model 1 was adjusted for baseline CRP and baseline SBP. Model 2 was additionally adjusted for age at baseline (continuous), gender, race (non-Hispanic white/non-Hispanic black/other races), anti-hypertensive medication use at baseline (yes/no), and statin use at baseline (yes/no). Model 3 was further adjusted for common RA treatments at baseline - TNFi, MTX, and leflunomide, which has a known association with elevated BP [27]. The potential effect of corticosteroids was also assessed by further adjusting the model for corticosteroid use. The same methods were applied to examine the associations between change in CRP and change in DBP, PP, and MAP.

Sensitivity analysis was performed by trimming extreme measurements of CRP (< 0.5% and > 99.5%) in both cohorts to prevent the strong influence of a small number of extreme values. Covariate data including age, gender, race, baseline anti-hypertensive medication use, and baseline statin use were extracted in both outpatient populations and NHANES.

To determine potential causes of elevated CRP in the outpatient population, we randomly selected 50 subjects with CRP in the highest decile. We reviewed medical records on or near the date of the elevated CRP and annotated the cause of the elevated CRP as attributed by the treating physician.

For all statistical analyses, a two-sided *P* value <0.05 was considered to be statistically significant. All data analyses were performed using SAS 9.4 (SAS Institute Inc., Cary, NC, USA) and R version 3.2.2 (R Development Core Team) statistical packages. This study protocol was approved by the Partners Healthcare Institutional Review Board.

## Results

A total of 24,325 subjects were included in the outpatient population, of whom 1811 had RA (RA outpatient population) and 22,514 did not (non-RA outpatient population). We studied 5561 subjects from the NHANES cohort (Table 1). Subjects with RA were generally older with mean age 60.4 years, compared to 54.0 years in the non-RA outpatient population and 47.9 years in NHANES. The median CRP was 3.4 mg/L (range 0.20–126.90 mg/L) in the RA outpatient population, 2.2 mg/L (range 0.10–416.20 mg/L) in the non-RA outpatient population, and 0.18 mg/L (range 0.01 to 18.01 mg/L) in NHANES. Additional characteristics for the RA population include 51.49% anti-cyclic citrullinated peptide (anti-CCP) positive, 41.25% on MTX, and 22.25% on TNFi.

**Table 1:** Clinical characteristic of subjects in the RA outpatient population, non-RA outpatient population and the general population (NHANES)

Characteristic	Outpatient population N = 24,325		NHANES* N = 5561
	RA N = 1811	Non-RA N = 22,514	
Age, years, mean (SD)	60.39 (13.97)	54.00 (15.55)	47.88 (18.49)
Female (%)	80.18	61.03	50.46
Ethnicity (%)			
Non-Hispanic white	80.51	80.40	48.48
Non-Hispanic black	5.63	5.46	16.90
Others	13.86	14.14	34.62
Anti-hypertensive medication use (%)	23.47	25.38	30.30
Statin medication use (%)	10.38	12.92	17.59
CRP, mg/L, median (IQR)	3.40 (1.40, 9.00)	2.20 (0.90, 5.80)	0.18 (0.07, 0.43)
SBP, mmHg, mean (SD)	129.92 (17.12)	126.48 (16.81)	122.14 (18.26)
DBP, mmHg, mean (SD)	75.62 (11.47)	76.07 (15.66)	69.30 (11.92)
PP, mmHg, mean (SD)	54.30 (14.57)	50.41 (17.94)	52.83 (17.51)
MAP, mmHg, mean (SD)	93.72 (11.76)	92.87 (13.65)	86.91 (11.74)

*RA* rheumatoid arthritis, *NHANES* National Health and Nutrition Examination Survey, *CRP* C-reactive protein, *SBP* systolic blood pressure, *DBP* diastolic blood pressure, *PP* pulse pressure, *MAP* mean arterial pressure

### Associations between CRP and BP in the RA and non-RA outpatient populations and NHANES

**Fig. 1:**
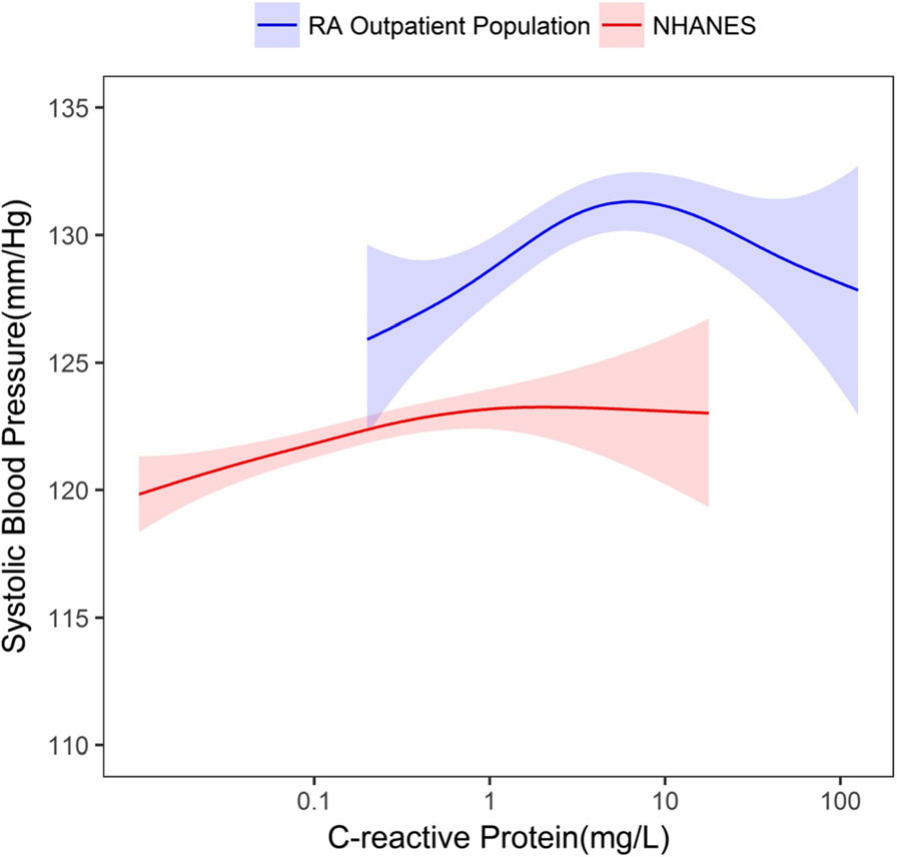
The relationship between C-reactive protein levels (CRP) and systolic blood pressure with 95% confidence intervals, in the rheumatoid arthritis (RA) outpatient population and the general population (National Health and Nutrition Examination Survey (NHANES)). RA outpatient population CRP range 0.20–126.90 mg/L; NHANES CRP range 0.01–18.01 mg/L

**Fig. 2:**
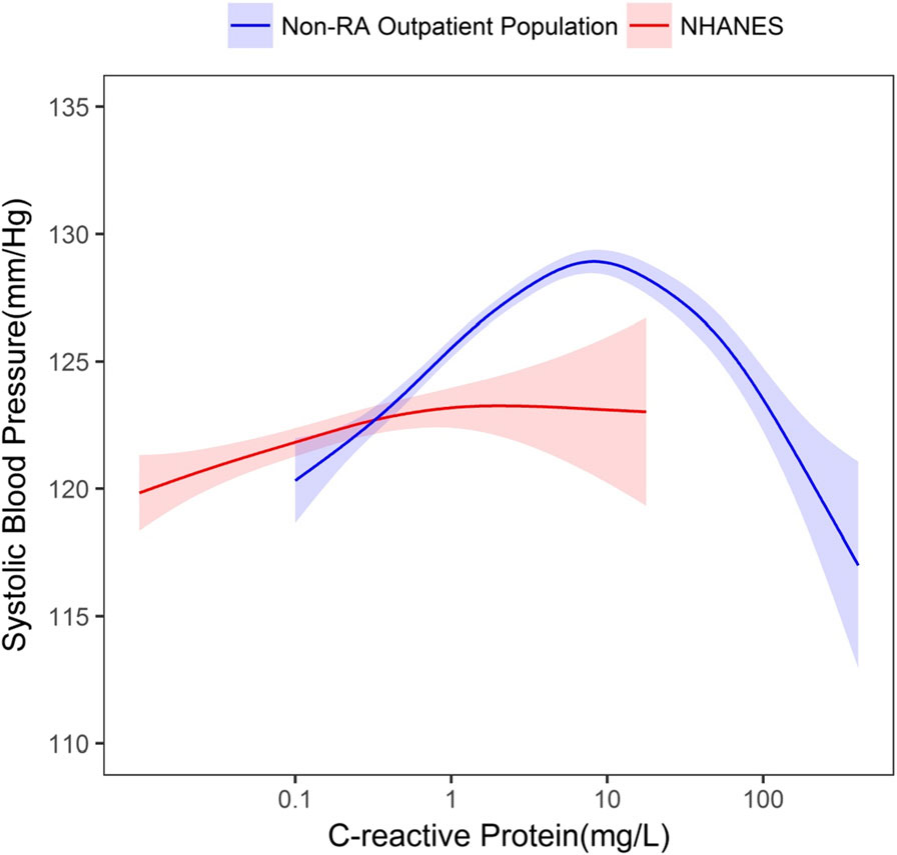
The relationship between C-reactive protein (CRP) levels and systolic blood pressure with 95% confidence intervals, in the non-rheumatoid arthritis (RA) outpatient population and the general population (National Health and Nutrition Examination Survey (NHANES)). Non-RA outpatient population CRP range 0.10–416.20 mg/L; NHANES 0.01–18.01 mg/L

The ranges of CRP were 0.20−126.90 mg/L in the RA outpatient population, 0.10–416.20 mg/L in the non-RA outpatient population, and 0.01 –18.01 mg/L in NHANES. In both the RA and non-RA outpatient populations, we observed an inverse U-shaped association between CRP and SBP (Figs. 1 and 2).

In the RA outpatient population, a positive association was observed between CRP and SBP in subjects with CRP < 5.94 mg/L. We observed an inverse relationship between CRP and SBP at CRP ≥ 5.94 mg/L **(**Fig. 1**)**. A similar relationship was observed between CRP and SBP in the non-RA outpatient population. A positive relationship was observed between CRP and SBP until CRP levels reached 8.39 mg/L. There was an inverse relationship between CRP and SBP at CRP ≥ 8.39 mg/L. In comparison, within NHANES, we observed a positive association between CRP and SBP.

The relationships between CRP and PP also differed in the RA and non-RA outpatient populations compared to NHANES, whereby there was an inverse relationship between CRP and PP after a threshold level of CRP was reached **(**Additional file 1: Figure S1B and Additional file 2: Figure S2B). The relationships between CRP and DBP and MAP were qualitatively more similar across the RA, non-RA and the general population cohorts (Additional file 1: Figure S1A and S1C, Additional file 2: Figure S2A and S2C). Findings from the sensitivity analysis with trimming of extreme measurements of CRP were similar to the main analysis (Additional file 3: Figure S3 and Additional file 4: Figure S4).

RA and other inflammatory arthritides (e.g. psoriatic arthritis, gout) comprised the largest percentage of subjects with CRP in the highest decile (34%), followed by infection (22%). Connective tissue diseases, including systemic lupus erythematosus, Sjogren’s syndrome, and myositis, were attributed by the treating rheumatologist to the elevated CRP in 10% of cases. Inflammatory bowel disease was attributed as the cause of the elevated CRP in 8% of cases and the etiology of the elevated CRP could not be determined in 14% of subjects.

### Longitudinal association between CRP and BP in RA

We identified 355 patients with RA from the RA outpatient population who had CRP changes defined as ≥ 10 mg/L or ≤ − 10 mg/L at ≥ 2 consecutive time points between 1 January 2009 and 1 June 2012. The median baseline CRP in this population was 9.7 (25th percentile 3.4, 75th percentile 25.4) mg/L. The mean SBP was 131.2 mmHg (SD 18.4) and DBP was 75.9 mmHg (SD 11.9). Of the 355 subjects with RA, 80.3% were female and 78.9% were white. The mean temporal interval between two time points in which CRP and BP were concomitantly recorded was 0.41 year (SD: 0.40). At baseline, 27.6% subjects were on anti-hypertensive medications, 10.7% on statin, 38.3% on MTX, 27.3% on a TNFi, and 15.2% on leflunomide.

Among patients with RA with significant inflammation change, as defined by CRP ≥ 10 mg/L, we observed a significant inverse association between the change in CRP and change in BP. Every 10 mg/L increase in CRP was associated with a 0.38-mmHg reduction in SBP. This relationship remained the same after adjusting for age, gender, race, anti-hypertensive medication, and statin use. As well, this relationship remained the same after additionally adjusting for potent immunomodulators such as TNFi, MTX, and leflunomide (Table 2). The association between CRP and SBP also remained the same after adjusting for corticosteroid use (β -0.38, 95% CI − 0.66, − 0.10, *p* = 0.007). An increase in CRP was also associated with reductions in DBP, PP, and MAP (Additional file 5: Table S1).

**Table 2:** Association between change in C-reactive protein (CRP) (per 10 mg/L) and change in systolic blood pressure (SBP) (per mmHg) in patients with rheumatoid arthritis with significant changes in inflammation

Models*	Change in SBP per 10 mg/L increase in CRP	
	Coefficient (95% CI)	*P* value
Model 1	− 0.38 (− 0.66, − 0.11)	0.007
Model 2	− 0.38 (− 0.66, − 0.11)	0.007
Model 3	− 0.38 (− 0.66, − 0.10)	0.007

*Model 1, adjusted for baseline CRP level and SBP; model 2, additionally adjusted for age (continuous), gender (male/female), race (non-Hispanic white/non-Hispanic black/other races), anti-hypertensive medication use (yes/no), and statin medication use (yes/no); model 3, additionally adjusted for tumor necrosis factor inhibitors medication use (yes/no), leflunomide use (yes/no), and methotrexate medication use (yes/no)

## Discussion

In summary, our study corroborates previous reports of a positive linear association between CRP and SBP. However, we found that this relationship was true only in the range of CRP typically observed in the general population. At higher CRP levels, we observed an inverse association between CRP and SBP. This inverse relationship was present not only in RA, but also in the non-RA outpatient population. Thus, previous studies on the association between inflammation and SBP may have shed light on only half the relationship. Studying a broader range of CRP levels in subjects allowed us to fill in information on the relationship between inflammation and BP between the two extremes, “normal” and those with sepsis. Studying data in a large population allowed us to identify this relationship.

Based on the cross-sectional study, a subject with CRP≥ 10mg/L would lie at the right side of the inverse U-shaped curve in Fig. 1. Based on the cross-sectional data, a reduction in inflammation would be associated with an increase in SBP. Indeed, this relationship was confirmed in the longitudinal analysis, where a reduction in CRP was associated with a modest increase in SBP. The association remained significant after adjusting for potential confounders including age, gender, and common RA treatments.

These findings may explain the absence of association between CRP and the outcome of hypertension in RA in a previous study [28]. The previous study used a binary outcome for hypertension, which would not detect a non-linear relationship between CRP and BP. Our findings are also consistent with current knowledge on the relationship between sepsis, an extreme form of inflammation, and BP. Uncontrolled inflammation in sepsis is linked with endothelial dysfunction and hypotension [29, 30]. Treatment and resolution of sepsis is associated with normalization of BP. Based on our data, we observed a similar though less severe pattern in subjects with RA, and in non-RA subjects with evidence of inflammation defined as elevated CRP. While there are studies on BP in sepsis, to our knowledge, there were no studies examining subjects in this intermediate group with mild to moderate CRP elevation.

The significance of our findings relates more to population health rather CV risk management at the individual level. In a recent study using data from the Atherosclerosis Risk in Communities study, investigators found that a 1-mmHg reduction in SBP was associated with substantial reduction in cardiovascular events at the population level [31]. Thus, the modest changes in SBP observed with changes in CRP may influence CV risk among subjects with inflammatory diseases. Taken in a larger context, the results of this study suggest a similar phenomenon to the lipid paradox in RA [32]. In the lipid paradox, lower lipids have not been associated with lower CV risk. Patients with RA with a reduction in inflammation, which is considered protective against CV risk, also experience an increase in low-density lipoprotein cholesterol (LDL-C) [10, 33], a marker of increased CV risk. In this study, reduction in inflammation (suggesting lower CV risk) was associated with modest increases in BP (suggesting higher CV risk). Thus, inflammation may be modifying the relationship between traditional CV risk factors, adding blood pressure to the list of factors already known to be altered in RA, including lipids and insulin resistance [34, 35].

Additionally, we observed that compared to the general population, the RA population on average had higher BP, at any given CRP level. While the mean BP at most CRP levels would not reach the threshold for classification as hypertension [36], these modest differences in SBP may also explain the known elevated risk for CVD in RA compared to the general population. The higher SBP in the outpatient population is likely due to a higher prevalence of hypertension in a clinic-based cohort compared to the general population.

The influences of inflammation on traditional CV risk factors may partially explain the underestimation of CV risk observed in several studies [37–39]. From the management perspective, this study adds to the current data on the importance of assessing CV risk among subjects with RA and those with other inflammatory diseases, when their disease is well-controlled. Assessing CV risk during active inflammatory disease may lead to measurements of both lipids and blood pressures that are lower than if the patients were in low disease activity or remission.

Limitations of this study include studying only patients who had CRP measured as part of routine care. As CRP is measured when a clinician is concerned about inflammation, the analysis likely includes the majority of patients with any type of inflammatory condition, which was a goal of the study. While CRP is only one of many markers of inflammation, it has been widely adopted for use across multiple conditions and was selected because it is a clinically interpretable test. Additionally, we adjusted for additional factors that may confound the association between CRP and BP, but not all factors were available for inclusion in the models. We examined known major confounders including age, gender, race, and anti-hypertensive and RA treatments.

We note that there are two potential confounders where ascertainment can be suboptimal using EMR data: non-steroidal anti-inflammatory drugs (NSAIDs) and corticosteroids. NSAIDs were not included in our models because the majority of NSAID use is over the counter not requiring a prescription, and so analysis of prescriptions for NSAIDs may be biased [40, 41]. The anticipated effect of NSAID use is elevated CRP, regardless of CRP levels, which would attenuate the association between elevated CRP and lower SBP. Thus, we anticipate that NSAID use would generally bias our findings towards the null. We observed no difference in association between change in CRP and change in SBP after adjusting for corticosteroids.

Another potential concern was differences in treatment among subjects with RA who had increases or decreases in their CRP, explaining the inverse association between CRP and SBP at higher CRP levels. When examining baseline treatments, we observed a similar percentage of biologic disease-modifying anti-rheumatic drug use among subjects who experienced a decrease in CRP (12.4%) compared to that among subjects who experienced an increase in CRP (12.5%). Other potential confounders not measured were the duration and doses of therapy. Last, the study population was selected from a tertiary care center and may not be generalizable to other community-based centers.

While identifying mechanisms for our findings is beyond the scope of this study, one potential mediator of this association is endothelial function, which plays an important role in vasomotor function and regulation of BP [42]. Patients with RA have increased arterial stiffness, which is attributed to endothelial dysfunction [43]. Treatment with the potent RA anti-inflammatory agent, TNFi, is associated with a significant reduction in disease activity and significant improvements in endothelial dependent and independent vasodilation of the brachial artery in patients with RA [44]. There is also evidence to suggest that endothelial dysfunction is influenced by other inflammatory cytokines in addition to TNF [45]. Together the data suggest that inflammation may impact endothelial function, vasomotor function, and regulation of BP. In line with these findings, we observed an inverse U-shaped association between CRP and PP, where PP is considered closely associated with endothelial function [46].

## Conclusions

In conclusion, our study suggests that CRP, used as a marker of inflammation, has a biphasic relationship with SBP when examining the relationship across the range of CRP levels observed in RA and in the outpatient setting. At lower levels of inflammation typically observed in the general population, an increase in CRP is associated with an increase in SBP. In RA and other states where a patient has elevated levels of inflammation, the relationship changes, such that CRP is associated with a decrease in SBP. When inflammation is mitigated, the relationship normalizes such that BP may increase as inflammation decreases, until general population levels of CRP are reached. While the changes in SBP were modest, they highlight a new relationship between CRP and BP not characterized previously. From the population health perspective, modest changes in SBP can have a substantial impact on CV risk. The pathways mediating these changes remains to be seen, however there is evidence to suggest endothelial function may play an important role. These data suggest a need to reevaluate our current approach to interpreting BP and CV risk in the setting of active inflammation in RA and other conditions, and the need for a better understanding of the pathways linking inflammation and BP regulation. Future studies in cohorts with detailed data on both inflammation and CV risk factors and outcomes are needed to determine the clinical significance of changes in inflammation on blood pressure and overall CV risk.

## Additional files

Additional file 1: **Figure S1.** The relationship between C-reactive protein levels (CRP) and diastolic blood pressure (A), pulse pressure (B), and mean arterial pressure (C) with 95% confidence intervals, in the RA outpatient population and general population (NHANES). RA, rheumatoid arthritis; NHANES, National Health and Nutrition Examination Survey. (PDF 1475 kb)

Additional file 2: **Figure S2.** The relationship between C-reactive protein levels (CRP) and diastolic blood pressure (A), pulse pressure (B), and mean arterial pressure (C) with 95% confidence intervals, in the non-RA outpatient population and the general population (NHANES). RA, rheumatoid arthritis; NHANES, National Health and Nutrition Examination Survey. (PDF 1361 kb)

Additional file 3: **Figure S3.** The relationship between C-reactive protein levels (CRP) and systolic blood pressure with 95% confidence intervals, in the RA outpatient population and the general population (NHANES) with trimming of extreme measurements of CRP (< 0.5% and > 99.5%). RA outpatient population CRP range 0.20–92.40 mg/L; NHANES CRP range 0.02–4.22 mg/L. RA, rheumatoid arthritis; NHANES, National Health and Nutrition Examination Survey. (PDF 476 kb)

Additional file 4: **Figure S4.** The relationship between C-reactive protein levels (CRP) and systolic blood pressure with 95% confidence intervals, in the non-RA outpatient population and general population (NHANES) with trimming of extreme measurements of CRP (< 0.5% and > 99.5%). Non-RA outpatient population CRP range 0.10–142.20 mg/L; NHANES CRP range 0.02–4.22 mg/L. RA, rheumatoid arthritis; NHANES, National Health and Nutrition Examination Survey. (PDF 471 kb)

Additional file 5: **Table S1.** Association between change in C-reactive protein (CRP) (per 10 mg/L) and change in diastolic blood pressure (DBP), pulse pressure (PP), and mean arterial pressure (MAP) (per mmHg) in patients with rheumatoid arthritis with significant changes in inflammation. (DOCX 17 kb)

## Abbreviations

BP: Blood pressure
BWH: Brigham and Women’s Hospital
CRP: C-reactive protein
CV: Cardiovascular
CVD: Cardiovascular disease
DBP: Diastolic blood pressure
EMR: Electronic medical record
MAP: Mean arterial pressure
MGH: Massachusetts General Hospital
MTX: Methotrexate
NHANES: National Health and Nutrition Examination Survey
NSAIDs: Non-steroidal anti-inflammatory drugs
PP: Pulse pressure
RA: Rheumatoid arthritis
RPDR: Research Patient Data Repository
TNFi: Tumor necrosis factor inhibitors
