# Correction to: Association between inflammation and systolic blood pressure in RA compared to patients without RA

**DOI:** 10.1186/s13075-019-1940-9

**Published:** 2019-07-08

**Authors:** Zhi Yu, Seoyoung C. Kim, Kathleen Vanni, Jie Huang, Rishi Desai, Shawn N. Murphy, Daniel H. Solomon, Katherine P. Liao

**Affiliations:** 1 Department of Epidemiology, Johns Hopkins Bloomberg School of Public Health, Baltimore, MD USA; 2 Department of Biostatistics, Johns Hopkins Bloomberg School of Public Health, Baltimore, MD USA; 3 Division of Rheumatology, Allergy and Immunology, Brigham and Women’s Hospital, Boston, MA 02115 USA; 4 Division of Pharmacoepidemiology and Pharmacoeconomics, Brigham and Women’s Hospital, Boston, MA USA; 5 Research Computing, Partners HealthCare, Charlestown, MA USA; 6 Laboratory of Computer Science, Massachusetts General Hospital, Boston, MA USA; 7 Department of Biomedical Informatics, Harvard Medical School, Boston, MA USA

**Correction to: Arthritis Res Ther**

**https://doi.org/10.1186/s13075-018-1597-9**

Following publication of the original article [1], it has come to our attention that we did not appropriately convert the units for CRP from the National Health and Nutrition Examination Survey (NHANES) from mg/dL to mg/L. In this study, NHANES was considered the general population cohort for this study; interestingly, the correction resulted in changes to a broader range of CRP values in NHANES from 0.01 to 18.0 mg/dL to 0.10 to 180.0 mg/L.

The overall results did not change the message for this study. We continued to observe a general positive association between CRP and blood pressure (BP) in all three groups, RA, non-RA and NHANES. For RA and non-RA this relationship held until a CRP level of approximately 7 mg/L when an inverse association was observed between CRP and systolic BP. In NHANES, we also observed a similar association of higher CRP associated with SBP until approximately 7 mg/L when the there is no association between CRP and SBP. In NHANES, 15% have a CRP above 7 mg/L compared to 32% in the RA and 21% non-RA cohorts. Thus, with the smaller percentage of subjects with a CRP above 7 mg/L in NHANES, there was potentially less power to detect an association.

Line-by-line corrections of our results are given below:

1. Results, page 3, last sentence in the right column should read: “…in the non-RA outpatient population, and 1.80 mg/L (range 0.10 to 180.10 mg/L).”
2. Results, page 4, 2nd paragraph, first sentence. The range of CRP was “0.10-180.10 mg/L in NHANES.”
3. Results, page 4, 3rd paragraph. “In comparison, within NHANES, we generally observed a positive association between CRP and SBP also until 7mg/dL when there is no association between CRP and SBP.”
4. Results, page 4, 2nd column, last sentence of the second paragraph, “Findings from the sensitivity analysis showed a general linear association between CRP and SBP in NHANES after trimming of the CRP outliers; this finding is consistent with previous studies in the general population. For RA and non-RA where higher levels are typically observed, the relationships were similar to the main analyses.”
5. In Table 1, the median (IQR) of CRP in NHANES should be 1.80 (0.70, 4.30) mg/L.
6. Figure 1 The relationship between C-reactive protein levels (CRP) and systolic blood pressure with 95% confidence intervals, in the rheumatoid arthritis (RA) outpatient population and the general population (National Health and Nutrition Examination Survey (NHANES)). RA outpatient population CRP range 0.20–126.90 mg/L; NHANES CRP range 0.10–180.10 mg/L (attached)
7. Figure 2 The relationship between C-reactive protein (CRP) levels and systolic blood pressure with 95% confidence intervals, in the non-rheumatoid arthritis (RA) outpatient population and the general population (National Health and Nutrition Examination Survey (NHANES)). Non-RA outpatient population CRP range 0.10–416.20 mg/L; NHANES 0.10–180.10 mg/L (attached)
8. We have also updated the supplementary figures to reflect the updated data:

**Fig. 1:**
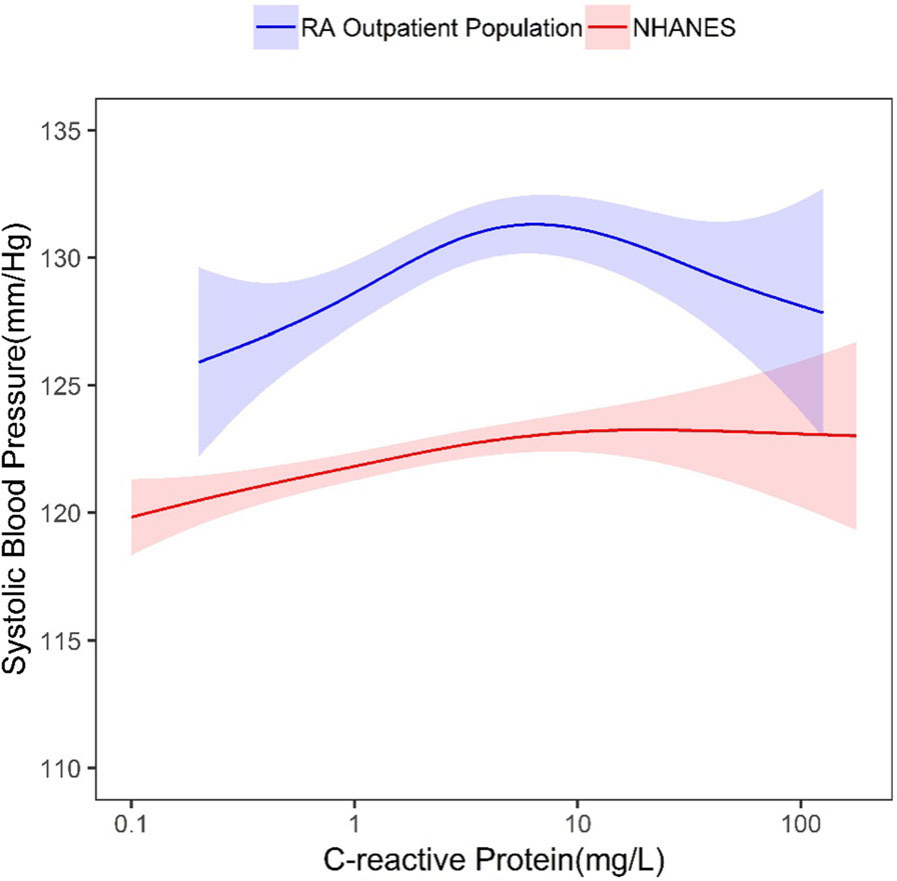
The relationship between C-reactive protein levels (CRP) and systolic blood pressure with 95% confidence intervals, in the rheumatoid arthritis (RA) outpatient population and the general population (National Health and Nutrition Examination Survey (NHANES)). RA outpatient population CRP range 0.20–126.90 mg/L; NHANES CRP range 0.10–180.10 mg/L

**Fig. 2:**
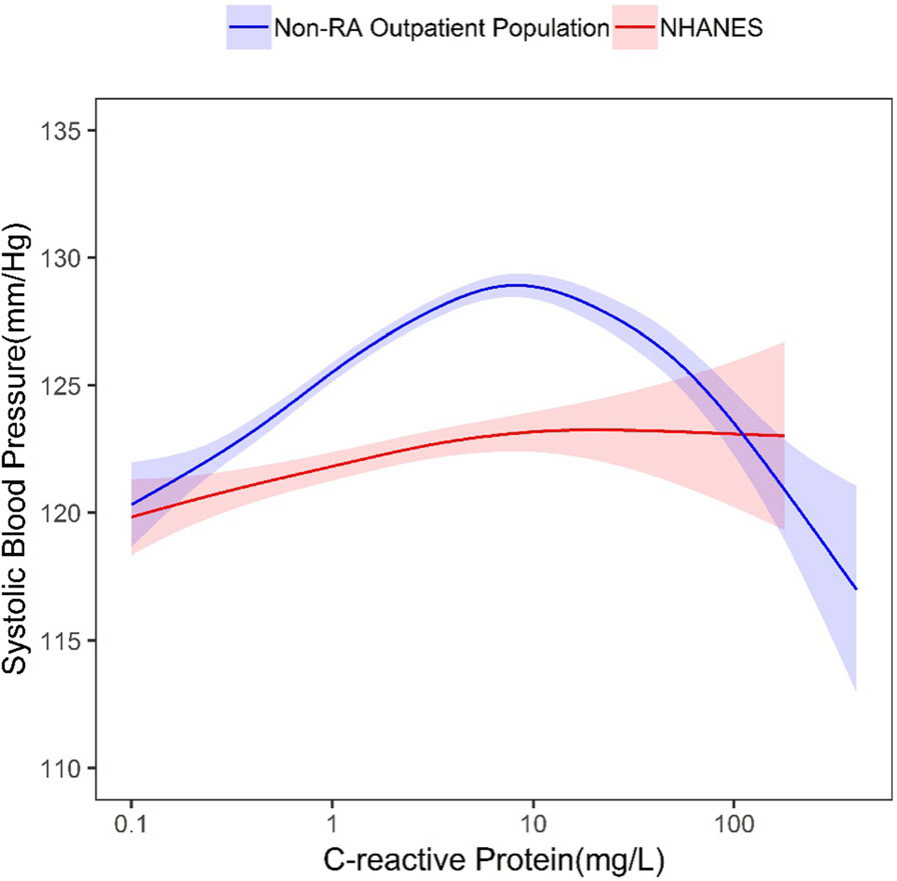
The relationship between C-reactive protein (CRP) levels and systolic blood pressure with 95% confidence intervals, in the non-rheumatoid arthritis (non-RA) outpatient population and the general population (National Health and Nutrition Examination Survey (NHANES)). Non-RA outpatient population CRP range 0.10–416.20mg/L; NHANES 0.10–180.10 mg/L

## Additional files

Additional file 1: **Figure S1.** The relationship between C-reactive protein levels (CRP) and diastolic blood pressure (A), pulse pressure (B), and mean arterial pressure (C) with 95% confidence intervals, in the RA outpatient population and general population (NHANES). RA, rheumatoid arthritis; NHANES, National Health and Nutrition Examination Survey. (PDF 108 kb)

Additional file 2: **Figure S2.** The relationship between C-reactive protein levels (CRP) and diastolic blood pressure (A), pulse pressure (B), and mean arterial pressure (C) with 95% confidence intervals, in the non-RA outpatient population and the general population (NHANES). RA, rheumatoid arthritis; NHANES, National Health and Nutrition Examination Survey. (PDF 111 kb)

Additional file 3: **Figure S3.** The relationship between C-reactive protein levels (CRP) and systolic blood pressure with 95% confidence intervals, in the RA outpatient population and the general population (NHANES) with trimming of extreme measurements of CRP (< 0.5% and > 99.5%). RA outpatient population CRP range 0.20–92.40 mg/L; NHANES CRP range 0.20–42.20 mg/L. RA, rheumatoid arthritis; NHANES, National Health and Nutrition Examination Survey. (PDF 12 kb)

Additional file 4: **Figure S4.** The relationship between C-reactive protein levels (CRP) and systolic blood pressure with 95% confidence intervals, in the non-RA outpatient population and general population (NHANES) with trimming of extreme measurements of CRP (< 0.5% and > 99.5%). Non-RA outpatient population CRP range 0.10–142.20 mg/L; NHANES CRP range 0.20–42.20 mg/L. RA, rheumatoid arthritis; NHANES, National Health and Nutrition Examination Survey. (PDF 13 kb)
