# Supplementary material for: Association between inflammation and systolic blood pressure in RA compared to patients without RA
Source: Arthritis Res Ther. 2018 Jun 1;20:107. doi: 10.1186/s13075-018-1597-9 (PMC5984318; doi:10.1186/s13075-018-1597-9)
Supplement: Supplementary file 1 — Figure S1. The relationship between C-reactive protein levels (CRP) and diastolic blood pressure (A), pulse pressure (B), and mean arterial pressure (C) with 95% confidence intervals, in the RA outpatient population and general population (NHANES). RA, rheumatoid arthritis; NHANES, National Health and Nutrition Examination Survey. (PDF 1475 kb) [file 13075_2018_1597_MOESM1_ESM.pdf]

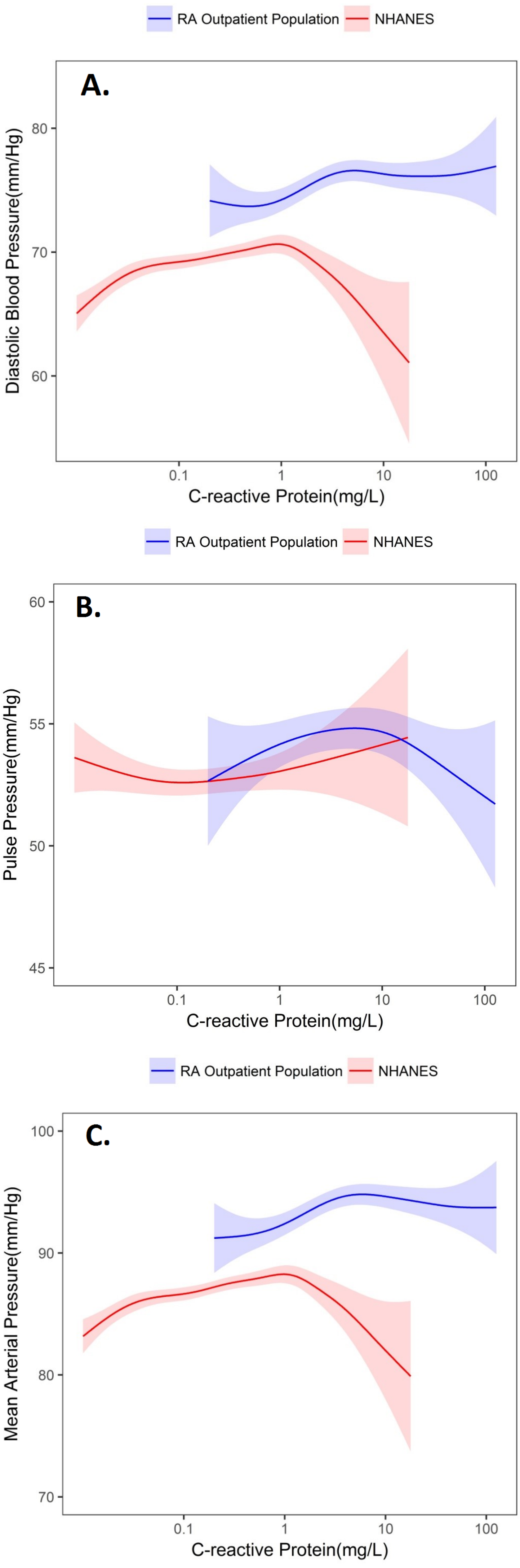

Figure S1. Associations between CRP with blood pressure measurements in the RA Outpatient Population compared to NHANES, specifically (A) diastolic blood pressure, (B) pulse pressure, and (C) mean arterial pressure.
