# Supplementary material for: Association between inflammation and systolic blood pressure in RA compared to patients without RA
Source: Arthritis Res Ther. 2018 Jun 1;20:107. doi: 10.1186/s13075-018-1597-9 (PMC5984318; doi:10.1186/s13075-018-1597-9)
Supplement: Supplementary file 3 — Figure S3. The relationship between C-reactive protein levels (CRP) and systolic blood pressure with 95% confidence intervals, in the RA outpatient population and the general population (NHANES) with trimming of extreme measurements of CRP (< 0.5% and > 99.5%). RA outpatient population CRP range 0.20–92.40 mg/L; NHANES CRP range 0.02–4.22 mg/L. RA, rheumatoid arthritis; NHANES, National Health and Nutrition Examination Survey. (PDF 476 kb) [file 13075_2018_1597_MOESM3_ESM.pdf]

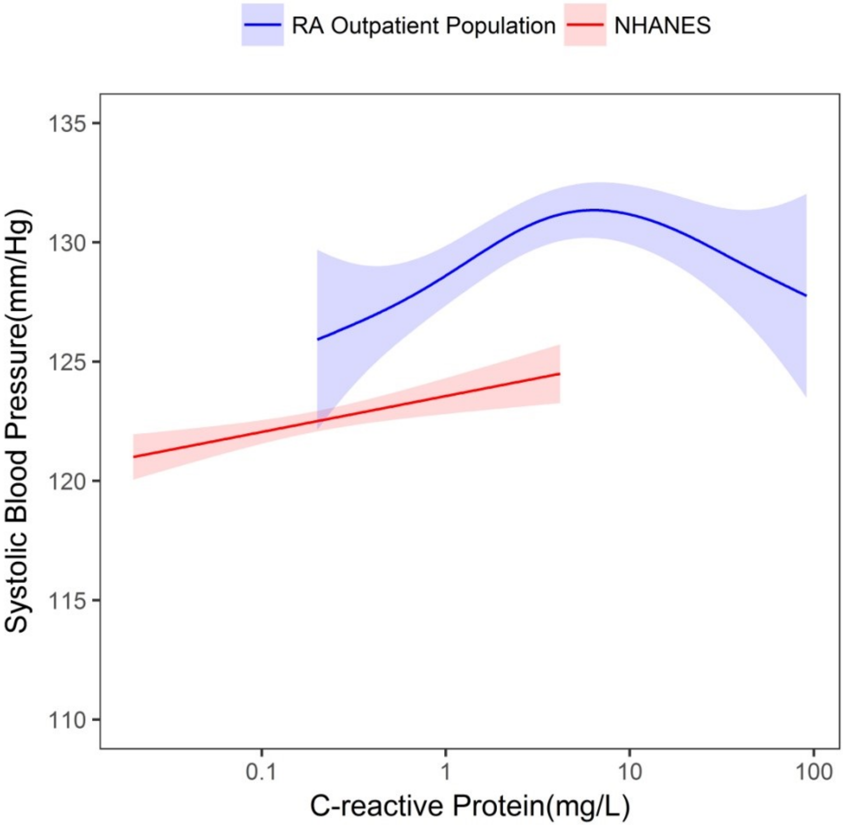


**Figure S3.** Sensitivity analysis demonstrating the relationship between CRP and systolic blood pressure (SBP) in the RA Outpatient Population compared to NHANES, where subjects with the 0.5% highest and lowest CRP levels were excluded.
