# Supplementary material for: Association between inflammation and systolic blood pressure in RA compared to patients without RA
Source: Arthritis Res Ther. 2018 Jun 1;20:107. doi: 10.1186/s13075-018-1597-9 (PMC5984318; doi:10.1186/s13075-018-1597-9)
Supplement: Supplementary file 5 — Table S1. Association between change in C-reactive protein (CRP) (per 10 mg/L) and change in diastolic blood pressure (DBP), pulse pressure (PP), and mean arterial pressure (MAP) (per mmHg) in patients with rheumatoid arthritis with significant changes in inflammation. (DOCX 17 kb) [file 13075_2018_1597_MOESM5_ESM.docx]

**Table S1.** Association between change in C-reactive protein (CRP) (per 10 mg/L) and change in diastolic blood pressure (DBP), pulse pressure (PP), and mean arterial pressure (MAP) (per mmHg) in rheumatoid arthritis patients with significant changes in inflammation.

| **Models*** | **Change in DBP**  **per 10mg/L increase in CRP** | | |  | **Change in PP**  **per 10mg/L increase in CRP** | | |  | **Change in MAP**  **per 10mg/L increase in CRP** | | |
| --- | --- | --- | --- | --- | --- | --- | --- | --- | --- | --- | --- |
|  | **Coefficient (95% CI)** |  | **P-value** |  | **Coefficient (95% CI)** |  | **P-value** |  | **Coefficient (95% CI)** |  | **P-value** |
| **Model 1** | -0.08 (-0.28, 0.11) |  | 0.400 |  | -0.32 (-0.57, -0.06) |  | 0.016 |  | -0.18 (-0.38, 0.01) |  | 0.059 |
| **Model 2** | -0.09 (-0.28, 0.11) |  | 0.391 |  | -0.31 (-0.57, -0.06) |  | 0.017 |  | -0.19 (-0.38, 0.01) |  | 0.058 |
| **Model 3** | -0.09 (-0.28, 0.11) |  | 0.394 |  | -0.31 (-0.57, -0.06) |  | 0.018 |  | -0.19 (-0.38, 0.01) |  | 0.058 |

*Model 1, adjusted for baseline CRP level and SBP; Model 2, additionally adjusted for age (continuous), gender (male / female), race (non-Hispanic white / non-Hispanic black / other races), anti-hypertensive medication use (yes / no), and statin medication use (yes / no). Model 3, additionally adjusted for tumor necrosis factor inhibitors medication use (yes / no), leflunomide use (yes/no), and methotrexate medication use (yes / no).
