# Supplementary figures and images for: Correction to: Association between inflammation and systolic blood pressure in RA compared to patients without RA
Source: Arthritis Res Ther. 2019 Jul 8;21:170. doi: 10.1186/s13075-019-1940-9 (PMC6615297; doi:10.1186/s13075-019-1940-9)

RA Outpatient Population NHANES

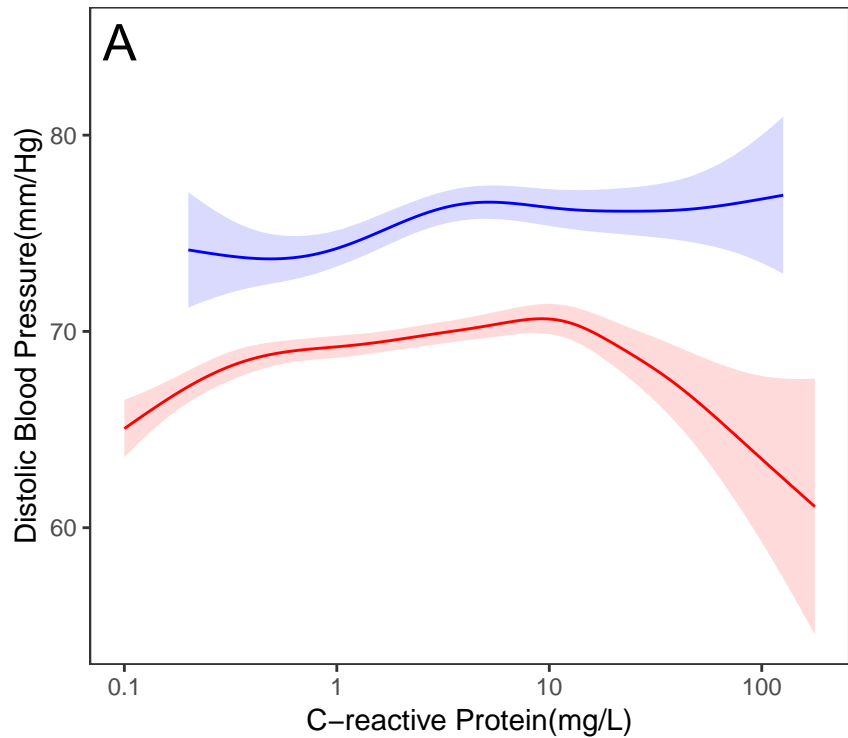

RA Outpatient Population NHANES

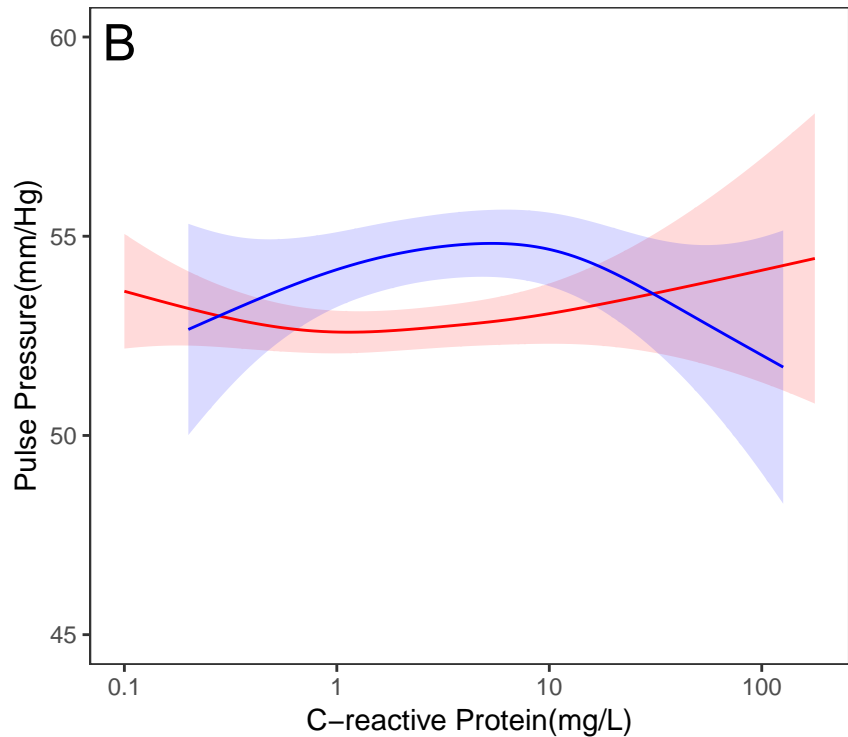

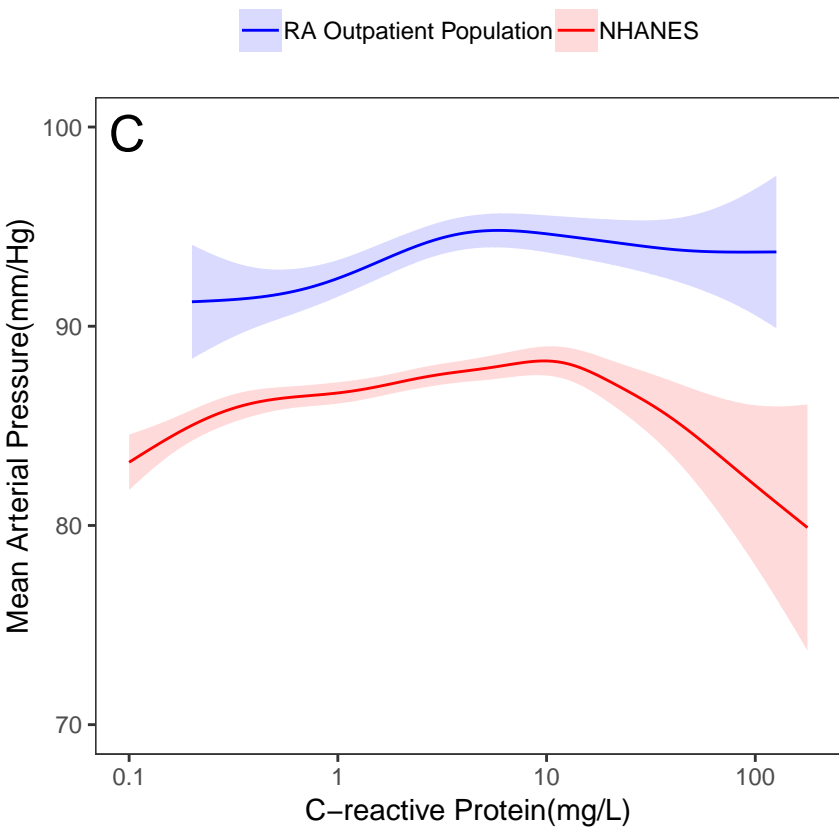

Supplement: Supplementary file 1 — Figure S1. The relationship between C-reactive protein levels (CRP) and diastolic blood pressure (A), pulse pressure (B), and mean arterial pressure (C) with 95% confidence intervals, in the RA outpatient population and general population (NHANES). RA, rheumatoid arthritis; NHANES, National Health and Nutrition Examination Survey. (PDF 108 kb) [file 13075_2019_1940_MOESM1_ESM.pdf]

RA Outpatient Population NHANES

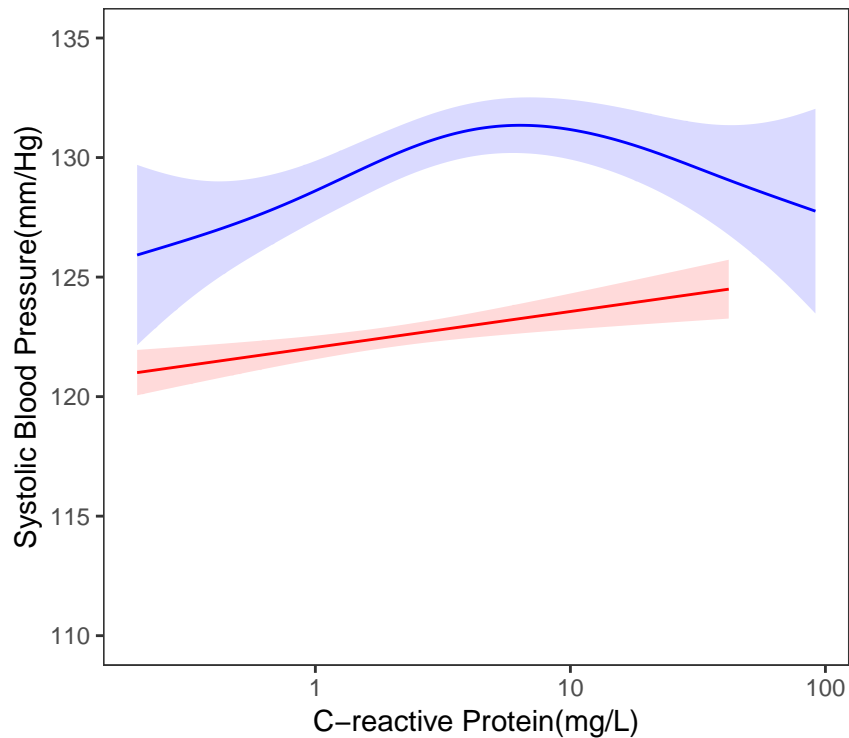

Supplement: Supplementary file 3 — Figure S3. The relationship between C-reactive protein levels (CRP) and systolic blood pressure with 95% confidence intervals, in the RA outpatient population and the general population (NHANES) with trimming of extreme measurements of CRP (< 0.5% and > 99.5%). RA outpatient population CRP range 0.20–92.40 mg/L; NHANES CRP range 0.20–42.20 mg/L. RA, rheumatoid arthritis; NHANES, National Health and Nutrition Examination Survey. (PDF 12 kb) [file 13075_2019_1940_MOESM3_ESM.pdf]

Non-RA Outpatient Population NHANES

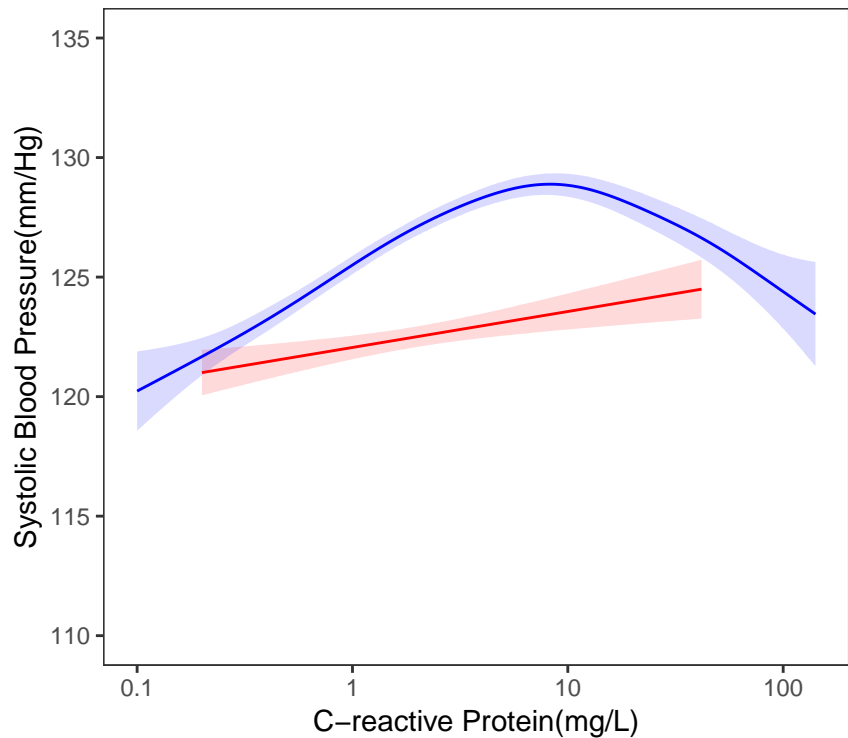

Supplement: Supplementary file 4 — Figure S4. The relationship between C-reactive protein levels (CRP) and systolic blood pressure with 95% confidence intervals, in the non-RA outpatient population and general population (NHANES) with trimming of extreme measurements of CRP (< 0.5% and > 99.5%). Non-RA outpatient population CRP range 0.10–142.20 mg/L; NHANES CRP range 0.20–42.20 mg/L. RA, rheumatoid arthritis; NHANES, National Health and Nutrition Examination Survey. (PDF 13 kb) [file 13075_2019_1940_MOESM4_ESM.pdf]
