# Supplementary material for: Correction to: Association between inflammation and systolic blood pressure in RA compared to patients without RA
Source: Arthritis Res Ther. 2019 Jul 8;21:170. doi: 10.1186/s13075-019-1940-9 (PMC6615297; doi:10.1186/s13075-019-1940-9)
Supplement: Supplementary file 2 — Figure S2. The relationship between C-reactive protein levels (CRP) and diastolic blood pressure (A), pulse pressure (B), and mean arterial pressure (C) with 95% confidence intervals, in the non-RA outpatient population and the general population (NHANES). RA, rheumatoid arthritis; NHANES, National Health and Nutrition Examination Survey. (PDF 111 kb) [file 13075_2019_1940_MOESM2_ESM.pdf]

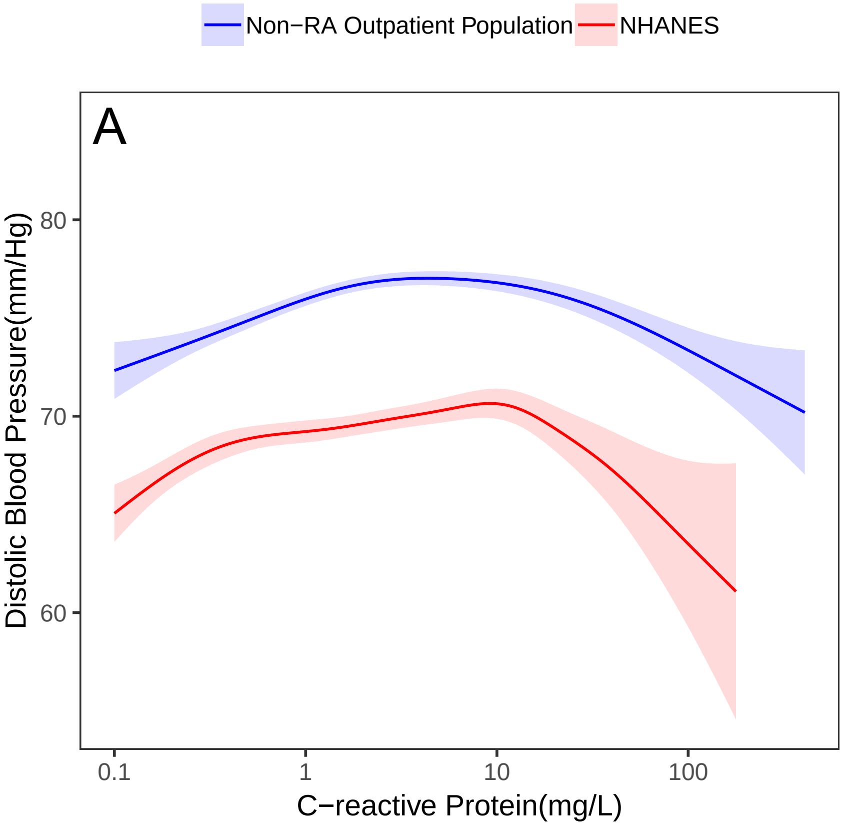

Non−RA Outpatient Population
NHANES
A
80
70
60
Distolic Blood Pressure(mm/Hg)
0.1
1
10
100
C−reactive Protein(mg/L)

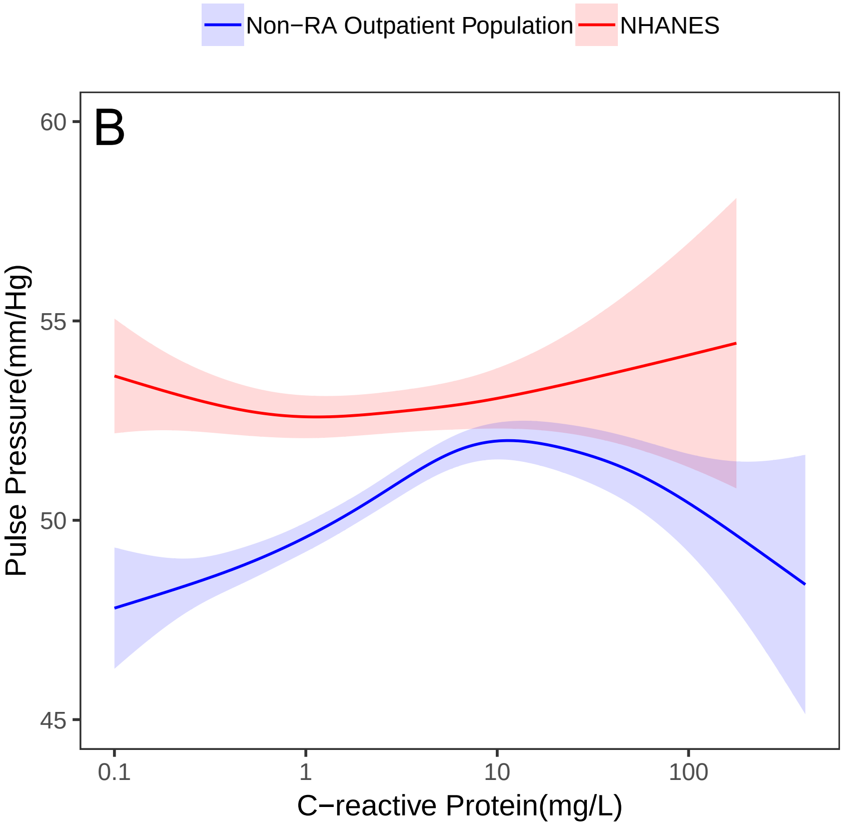
Non−RA Outpatient Population
NHANES
B
Pulse Pressure(mm/Hg)
60
55
50
45
0.1
1
10
100
C−reactive Protein(mg/L)

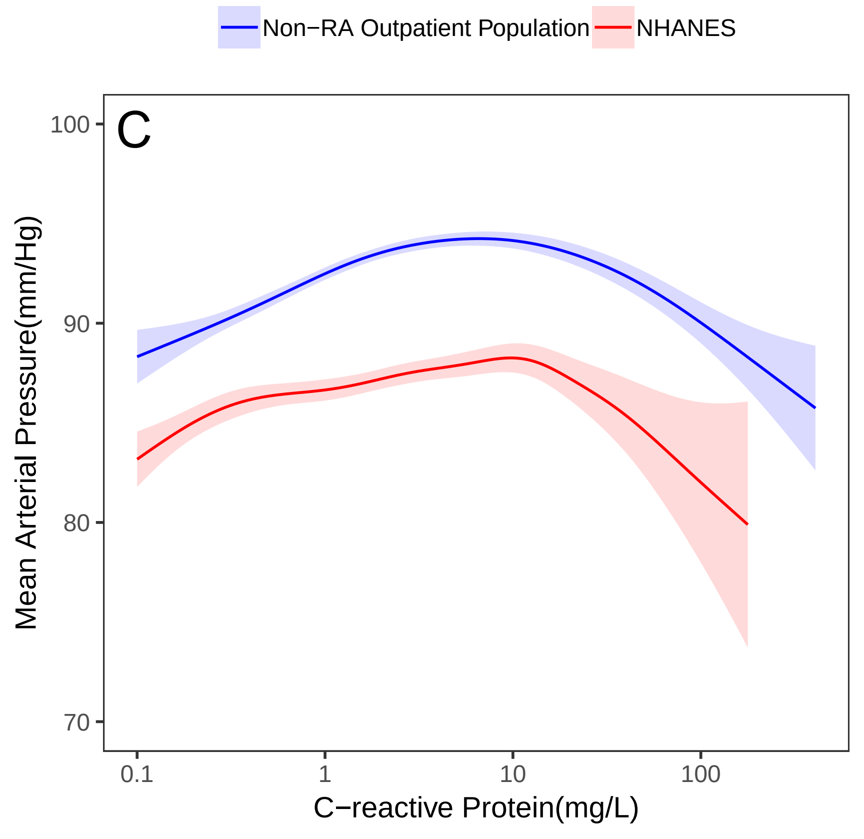
Non−RA Outpatient Population
NHANES
C
100
90
80
70
0.1
1
10
100
Mean Arterial Pressure(mm/Hg)
C−reactive Protein(mg/L)
